# Participation rate in cervical cancer screening in general practice related to the proximity of gynecology care facilities: A 3 year follow-up cohort study

**DOI:** 10.3389/fpubh.2022.955559

**Published:** 2022-10-17

**Authors:** François Quersin, Fanny Serman, Jonathan Favre, Michaël Rochoy, Axel Descamps, Elise Gers, Alain Duhamel, Claire Collins, Valérie Deken-Delannoy, Christophe Berkhout, Thibaut Raginel

**Affiliations:** ^1^Department of General Practice/Family Medicine, University of Lille, Lille, France; ^2^University of Lille, CHU Lille, Intern Promotion Division of the Research and Innovation Board, Lille, France; ^3^University of Lille, CHU Lille, EA 2694 – Public Health: Epidemiology and Quality of Care, Lille, France; ^4^Irish College of General Practitioners, Dublin, Ireland; ^5^University of Lille, CHU Lille, Service de Statistique, Evaluation Economique, Data-Management, Lille, France; ^6^Department of Primary Health and Interprofessional Care, University of Antwerp, Antwerp, Belgium; ^7^Normandy University, UniCaen, Inserm U 1086 “Anticipe”, Caen, France; ^8^Normandy University, UniCaen, Faculty of Health, Department of General Practice, Caen, France

**Keywords:** uterine cervical neoplasm, screening, general practice, primary healthcare, Ilot Regroupé pour l'Information Statistique (French smallest area for statistical data), delivery of health care, lower-layer super output areas

## Abstract

Cervical cancer screening (CCS) by Pap tests is mainly performed by gynecologists in France, but also by general practitioners (GPs) and midwives. The screening uptake is insufficient to reduce the incidence of cervical neoplasms. Our aim was to investigate the association between screening rates in patients listed with GPs and the distance between GPs' offices and gynecology facilities. The population of 345 GPs, and their 93,918 female patients eligible for screening over 3 years (2013–2015), were derived from the Health Insurance claim database. We estimated the socioeconomic level of the geographical area of GPs' offices using the European Deprivation Index (EDI). The proximity of gynecology facilities was calculated by computing their distance from GPs' offices (in order to adjust the proximity of gynecology facilities with EDI and performance of smears by the GP). The number of gynecologists within 5 km of a GP's office was associated with the CCS rate increasing by 0.31% for every unit increase in the density of gynecologists within 5 km (*p* < 0.0001). The close proximity of gynecology facilities was not significantly associated with screening uptake among female patients when the office of the GP where they were registered was settled in a deprived area.

## Introduction

According to the Global Cancer Observatory (GLOBOCAN) from 2018 estimating cancer data from 185 countries, cervical cancer (CC) was the fourth most common cancer in women worldwide, with a global age-standardized incidence rate (ASIR) of 13.1/100,000 women. This ASIR varied widely among countries ranging from <2 to 75/100,000 (1). In Europe between 2012 and 2018, the ASIR of CC varied from 13.4 to 13.9/100,000, and in France from 8.0 to 8.4/100,000, and the age-standardized mortality rate (ASMR) from 2.6 to 3.2/100,000, showing an increase after four decades of decrease (2, 3). In France, there were 2,920 new cases of CC and 1,117 related deaths in 2018 (4). In Northern France, the incidence rate is 10% higher compared to the country average (4).

CC is always preceded by neoplastic lesions with a long-lasting persisting evolution before reaching a cancerous stage. This offers the opportunity to prevent cancer by screening and early intervention. The classical screening test is the Papanicolaou-test (Pap-test) by cytologic examination of cervical smears, which requires a gynecological examination. To implement cervical cancer screening (CCS), French health authorities recommend a Pap-smear every 3 years in women between 25 and 29 years of age after two annual normal initial Pap-smears. Since 2019, the same authorities recommend a HPV test every 5 years between 30 and 65 years of age; in the case of positive test a cytology must be achieved. In the case of negative cytology, screening must occur again the next year following the same procedure (5). In France in 2017, CCS was “opportunistic” except in 13 departments testing an experimental organized screening. The screening participation rate is not in accordance with the recommended rate of 80% in the guidelines for women in the target ages, being insufficient for 51.6% of women or too frequent for 40.6% (5).

In high income countries, insufficiently screened women are mainly those who do not use the services of gynecologists for cultural or economic reasons: low level of education or income [consultations with a gynecologist being more expensive than those with a general practitioner (GP)], women with no children, having no partner or being post-menopausal (6). Most of these women have at least one encounter with their GP over 3 years. In France, 80% of targeted women have previously chosen to be screened by a gynecologist but their numbers are drastically decreasing (7). In French Flanders, 53.1% of GPs and more recently midwives also perform this procedure (8). The performance of smears by the GP or the female gender of the GP, described as positive factors for participation in CCS, do not increase the rates significantly (9). Socioeconomic environmental factors like the European Deprivation Index (EDI) appear significantly and independently associated with these rates, women dwelling in deprived areas being more often insufficiently screened or not screened at all (10). Another factor described as positive for participation in CCS is the proximity of the office of a gynecologist (11).

Our interest was to investigate the effect of the close proximity of the office of a gynecologist on the CCS participation rates. In our former publications (8–10), we acknowledged as main limitations a follow up period of 2 years and not controlling for the influence of the gynecology care facilities. These elements are considered in this paper.

## Materials and methods

### Study design

As at that time (2017), the recommended interval between two CCS smears was 3 years, a cohort study was undertaken based on a 3 year retrospective follow up of 93,918 female patients aged from 25 to 65 years and their 345 GPs coupled with a telephone survey.

### Setting

This study took place in primary care in French Flanders (Northern France). Data were collected from 2013/01/01 to 2015/12/31 from the Information System of the main mandatory Health Insurance claim database (SIAM) of French Flanders (CPAM). Telephone surveys with all the practicing GPs registered with the CPAM were carried out.

### Participants

Participants were the GPs listed on the registers of the CPAM. Inclusion required that the GPs were practicing in primary care over the 3 year period selected. GPs having another practice outside of primary care were excluded if they had <100 female patients declared on their patient lists, ruling out GPs with complementary medicine practices (for example homeopathy, acupuncture), other practices than primary care (for example sonography and angiology) and GPs with an unbalanced practice (recently established or nearing retirement). GPs who retired during the follow up period and those who refused to answer the telephone surveys were also excluded.

For the included GPs, we considered their female patient population aged from 25 to 65 years eligible for cervical cancer screening under French guidelines.

### Variables

The main outcome was the cervical cancer screening participation rate in the eligible female patient population of included GPs, measured by the refunding to female patients of cytological examination of cervical samples by the health insurance fund.

Working with claim databases where patients are anonymised and not traceable for regulatory ethic reasons (we only know their gender, their age between 25 and 65 years, the designation of their GP, and the reimbursement of a pap smear), it was not possible to compute the distance between the dwelling place of patients and offices of gynecologists. However, most of the patients are registered on the patient lists of their closest settled GP and share the same environmental characteristics (10). As a surrogate outcome of the distance between the dwelling place of patients and offices of gynecologists, we computed as our proximity indicator the density of the gynaecologists' offices around GPs' offices within 5, 10, 20, and 40 km. Thus, the predictor was the distance between the office of a gynecologist and each GP office. This variable was computed using geo-tracking of GP offices and the gynaecologists' offices.

The confounding variables on the GP level were the gender of the GP (recovered from the SIAM database) and the performance of vaginal samplings (as a binary variable) in the GP office based on telephone surveys as described in a former paper (8).

The European Deprivation Index (EDI) (12) was the socio-economic effect indicator utilized. The EDI is an ecological marker reflecting the individual deprivation experience of the general population in an area based on the census. The determination of EDI started from the construction of an individual deprivation indicator associated with both objective and subjective poverty and following the identification of the basic needs of people. This first part was undertaken using the European survey specifically dedicated to the study of deprivation (EU-SILC: European Union—Statistics on Income and Living Conditions), since there is no gold-standard of deprivation. It was then necessary to identify and dichotomize the variables available and coded in a similar way both at the individual level (EU-SILC) and in the census data. Variables associated with the individual deprivation indicator were then selected and weighted by multivariate logistic regression. The regression coefficients associated with these variables in the final model then became the weights of these 10 variables measured at the aggregate level in the ecological index: overcrowding, no access to a system of central or electrical heating, non-home owner, unemployment, foreign nationality, no access to a car, unskilled worker—farm worker, household with more than six persons, low level of education, single parent household. The EDI is then defined as the weighted sum of these 10 variables quantifying fundamental basic needs associated with both objective and subjective poverty, normalized to the national average and usually divided into quintiles (national or regional). Areas of reference were the smallest available statistical census units in France (IRIS) allowing for an infra-municipal study scale. Each GP surgery was assigned its IRIS and the EDI of the corresponding IRIS was computed. Elsewhere (10), we have demonstrated the strong association between the EDI and the CCS rate. The EDI has a mediation effect on the CCS uptake.

### Bias

The number of patients managed by the GP was not considered as we have demonstrated that it is not associated with the CC screening rate (8). The age of the GP has not been considered though it appears to be associated with the screening rate, as it is linked to the age of the patients, and young female patients are more likely to participate in cervical cancer screening compared to older patients (13, 14). Another reason is that young GPs are more often of female gender compared to older GPs, and the performance of smears is associated with the gender of the GP as demonstrated earlier (8), though without influence on CCS uptake in a multivariate analysis. The gender of the GP and the performance of smears therefore seemed to be sufficient substitution variables.

### Study size

This study was implemented on a complete population basis without sampling.

### Statistics and analysis

Continuous quantitative variables are expressed as mean ± standard deviation (SD), median [interquartile range (IQR)] and categorical variables are expressed as frequencies and percentages. In this study, there were two hierarchical levels for the data: the individual GP level (GP's gender and performance of smears, and the outcome “the cervical cancer screening participation rate among the GP's listed eligible female patients”) that were nested in the geographical level (variable EDI and number of gynecologists at a given distance) as the patients of GPs practicing in the same area (IRIS) share common characteristics. The association between the CCS rate and the distance from gynaecologists' offices was analyzed using a linear generalized hierarchical mixed model. This statistical model takes into account the hierarchical structure of the data. The analysis was performed without and with adjustment based on the characteristics of the GPs and the socioeconomic level considered as a mediator (EDI).

All statistical tests were two-sided and performed at the 0.05 level. Data were analyzed using the SAS software^®^ version 9.4 (SAS Institute, Cary, NC).

### Bioethics

The protocol of this trial is available on Clinical Trials under the reference NCT02749110. It was approved by the ethics committee North West III of Caen under the reference 2015-23, on 2016/03/02.

## Results

**Figure 1:**
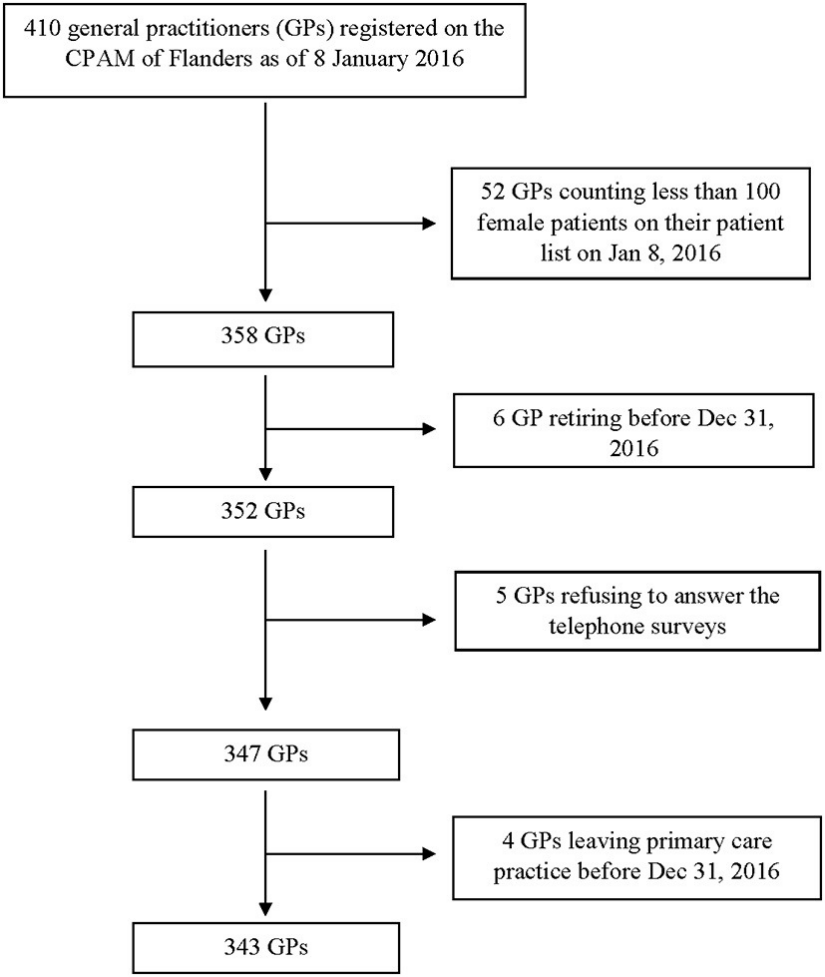
Flow chart.

Of the 410 GPs registered on the CPAM of Flanders, 52 were excluded as they had <100 female patients on their patient lists, six because they retired before the end of the study period, five because they refused to answer the telephone survey and two because they planned to suspend their activity as primary care practitioners, resulting in 343 included GPs (Figure 1).

Among the 343 GPs participating in this study, 269 (78.4%) were men, and 182 GPs (53.0%) performed smears. Characteristics of the listed patients per GP are described in Table 1 and shows the mean screening participation rate for female patients from 25 to 65 years during the 3 years was 50.1% (SD: 7.5%).

**Table 1:** Characteristics of the listed patients per GP.

**Characteristics**	**Mean (SD)**	**Median (IQR)**
Number of listed patients	702.9 (293.8)	666 (497–852)
Number of listed women	375.8 (158.5)	352 (266–451)
Number of screened women	145.4 (69.9)	134 (98–177)
Number of listed women 25–65 years	272.2 (123.1)	256 (185–329)
Number of screened women 25–65 years	136.5 (65.6)	126 (92–167)
Percentage of screened women 25–65 years	50.1% (7.5%)	50.6% (44.7–55.3)

SD, Standard deviation; IQR, Interquartile range.

**Figure 2:**
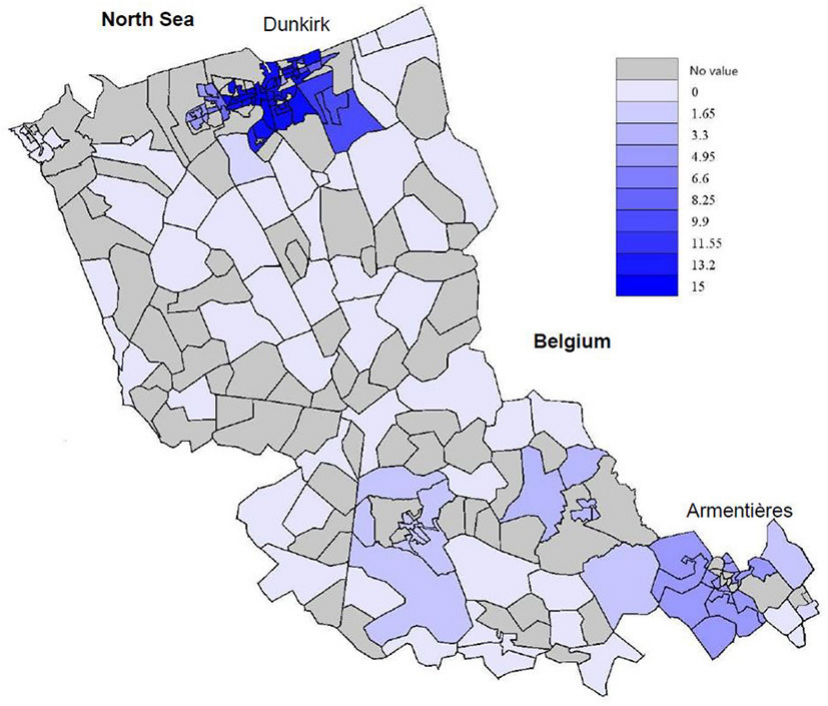
Density of gynecology care facilities within 5 km of a GP surgery on the territory of the CPAM of Flanders.

The mean number of gynecologists within 5 km of GP surgeries was 5.4 (SD 5.6, median 5, IQR 0–11), between 5 and 10 km was 3.1 (SD 6.2, median 1, IQR 0–3), between 10 and 20 km was 15.2 (SD 21.0, median 7, IQR 0–20) and between 20 and 40 km was 30.8 (SD 28.1, median 15, IQR 10–42) (Figure 2).

The association between the cervical screening rate and the distance from gynecology care facilities is shown in Table 2. The table presents the unadjusted model, the model adjusted for GP gender, and performance of smears by the GP and the model adjusted for GP gender, performance of smears by the GP and EDI. All models show the impact of the density of gynecologists within specified distances from the GP offices on the CCS rate with the greatest impact being the density within 5 km. The density of gynecologists within 20–40 km of GP surgeries had a smaller significant positive coefficient.

**Table 2:** Association between the cervical screening rate and the density of gynecologists.

	**Unadjusted model**		**Adjusted model** ^**^		**Adjusted model** ^***^	
**Characteristics**	Coefficient (95%CI)^*^	***p***	Coefficient (95%CI)^*^	***p***	Coefficient (95%CI)^*^	***p***
Density <5 km	0.184 (−0.016; 0.384)	0.07	0.203 (0.006; 0.400)	0.04	0.312 (0.158; 0.466)	<0.0001
Density 5–10 km	0.125 (−0.041; 0.291)	0.14	0.145 (−0.017; 0.307)	0.08	0.093 (−0.029; 0.215)	0.14
Density 10–20 km	−0.011 (−0.060; 0.037)	0.64	−0.014 (−0.062; 0.033)	0.55	−0.033 (−0.068; 0.000)	0.06
Density 20–40 km	0.086 (0.047; 0.125)	<0.0001	0.082 (0.044; 0.121)	<0.0001	0.074 (0.045; 0.104)	<0.0001

* Regression coefficient from linear mixed model with 95% confidence interval.

** Adjusted model with GP's gender, performance of smears by the GP.

*** Adjusted model with GP's gender, performance of smears by the GP and EDI.

We found a significant association between the density of gynecologists within 5 km of the GP's office and the cervical cancer screening participation rate after adjustment for these GP characteristics and the EDI, with the cervical screening rate increasing by 0.31% with every unit increase in the density of gynecologists within 5 km. When not adjusting for EDI, the density of gynecologists within 5 km of GP surgeries had no significant effect on the screening rate.

The density of gynecologists between 20 and 40 km also had a significant effect, with the cervical screening rate increasing by 0.09% with every unit increase in the density of gynecologists between 20 and 40 km. After adjusting for the GP's gender, practice of Pap-smears by the doctor and EDI, the association remained significant though the effect size was small.

## Discussion

### Main findings

In the analysis adjusting for EDI, we found that the density of gynecologists within 5 km of GP surgeries had the most significant positive regression coefficient with the CCS rate. We also found that the density of gynecologists within 20–40 km of GP surgeries had a smaller but still significant positive coefficient.

The higher the density of gynecologists within 5 km of GP surgeries, the higher the CCS rate: for each supplementary gynecologist, the screening rate was improved by 0.31%. When not adjusting for EDI, the density of gynecologists within 5 km of GP surgeries had no significant effect on the screening rate, reflecting the major influence of socioeconomic determinants on screening behavior. Thus, in disadvantaged areas (like French Flanders: EDI of 2.3 compared to the mean EDI of 0 for France), a higher number of gynecologists does not increase the screening rate in the overall population, unless erasing the influence of the deprivation factor. This reflects the fact that women from deprived areas are not likely to be managed by gynecologists while women from more favored areas are more likely to be so (15, 16).

### Study strengths and limitations

The claim database of the CPAM of Flanders is reliable and consistent, and the data extracted from this database for a duration of 3 years are considered trustworthy. A participation rate of 98% of the targeted GPs allows us to consider that our study was based on an entire, not sampled population. Including 345 GPs, their almost 94,000 female patients eligible for CCS, and the 149 gynecologists in the area who may have been consulted by these patients, confers to this study a solid internal validity.

Studies exploring the association between the density of gynecological care facilities and the CCS participation rate as the main outcome are scarce. No one has previously explored this association based on the ground distance between GPs and gynecologists. Our results match another French country-side study highlighting the same association by another method (11) strengthening the external validity of our finding.

In our previous publications, we acknowledged as limits a follow up of only 2 years (as CCS used to be triennial in France) and no consideration of gynecology care facilities as confounding factors (8–10). These limits have been addressed in the current paper.

This study only investigates the association between CCS rates and the distance from gynecologist offices to GP offices. The global screening rate in French Flanders of 50.2% is lower than the national rate of 62.3% (range 41.6–72.5%) (17). The density of GPs in French Flanders was slightly lower than in the rest of France (13 vs. 16/10 000) and is even lower now (retirement of GPs from the baby-boom generation). However, the density of gynecologists (2.7/10 000) in this area was not lower than in the rest of France, which does not explain under-screening and our findings regarding the highlighted association.

There are many different compulsory health insurance regimes in France depending on the occupational sector of the insured persons. We based our study on the claim database of the CPAM of Flanders representing 80% of insured persons. This means that we missed some occupational sectors like teachers or farmers. This can possibly be considered as a selection bias though there is no reason that the 20% of missed population substantially differs from the general population as described in other contexts (18). This does not diminish the external validity of our main result.

### Comparison to literature

The only former publications investigating this association are the above cited French study carried out by Araujo in 2010 (11), and another French study published by Barré in 2017 (19), which found that a lower CCS participation rate was associated with a lower density of gynecologists in the residence area, matching our findings. A third study, carried out by Grillo in 2012 in Paris, did not find any significant association between the density of GPs and gynecologists in the residence area of women and the CCS participation rates (20). However, no adjustment was performed to correct for the influence of the overall deprivation rate and the geographical area studied was smaller with more opportunities for public transport.

Profound changes in the mindset of women influenced by the social pressure of their deprived neighborhood will be necessary to enhance participation in CCS. The proximity of care facilities has little influence on enhancing screening participation in deprived areas unless community oriented primary care reaches out to concerned people (21–23). Education is probably the main solution to solve the lost opportunity associated with underscreening in deprived areas (24).

## Conclusion

Adjusting for our deprivation indicator (EDI), the density of gynecology care facilities within 5 km of a GP surgery, and to a lesser extent, within 20–40 km of a GP surgery, was significantly associated with a higher CC screening rate. When the effect of deprivation on the screening participation rate is erased by adjusting the model, the density of gynecology care facilities is linked to an increase of the CCS participation rate, meaning a potential decrease of CC. However, this effect is not noticeable when this adjustment is not made probably because women dwelling in deprived areas do not make use of services offered by gynecologists. The reasons women are not screened are complex and this certainly explains why medical demography alone cannot resolve inequalities and social disparities in participation in screening. This seemed to be confirmed by our models, despite its adjustment using the EDI (an aggregate index quantifying fundamental basic needs associated with both objective and subjective poverty). The current setting of midwifes in primary care practices might be a response to this situation that will have to be confirmed by further studies.

## Data availability statement

The raw data supporting the conclusions of this article will be made available by the authors, without undue reservation.

## Ethics statement

The studies involving human participants were reviewed and approved by Ethics Committee North West III of Caen under the reference 2015-23, on 2016/03/02. The patients/participants provided their written informed consent to participate in this study.

## Author contributions

Conceptualization and funding acquisition: JF, MR, ADu, VD-D, CB, and TR. Methodology, resources, and writing—review and editing: FQ, FS, JF, MR, ADu, EG, ADe, CC, VD-D, CB, and TR. Software and formal analysis: FQ, EG, and VD-D. Validation: JF, MR, EG, VD-D, CB, and TR. Investigation: FQ, FS, ADe, EG, VD-D, and CB. Data curation: EG, ADu, and VD-D. Writing—original draft: FQ and TR. Visualization: FQ, FS, JF, MR, ADe, CB, and TR. Supervision: CB. Project administration: EG, ADu, VD-D, and CB. All authors have read and agreed to the published version of the manuscript.

## Funding

This was an ancillary study of the PaCUDAHL-Gé trial sponsored by the University Hospital of Lille, which evaluates women's interest in cervical cancer screening using a device from their GP for self-collection of vaginal samples and HPV testing. The trial was funded by the French Ministry of Health (PREPS: LIC-14-14-0615, 2014/12/19).

## Conflict of interest

The authors declare that the research was conducted in the absence of any commercial or financial relationships that could be construed as a potential conflict of interest.

## Publisher's note

All claims expressed in this article are solely those of the authors and do not necessarily represent those of their affiliated organizations, or those of the publisher, the editors and the reviewers. Any product that may be evaluated in this article, or claim that may be made by its manufacturer, is not guaranteed or endorsed by the publisher.
